# Super refractory status epilepticus as an atypical presentation of Hashimoto’s encephalopathy

**DOI:** 10.1016/j.ebr.2026.100862

**Published:** 2026-03-16

**Authors:** Andres Chaponan-Lavalle, Jorge Alave, Fred Fernandez, Luciana Chacon Hermoza, Danny M. Barrientos-Iman, Roberto Chulluncuy-Rivas, Nelson Diaz

**Affiliations:** a Universidad Peruana de Ciencias Aplicadas, Lima, Peru; b Universidad Peruana Union, Lima, Peru; c Clinica Good Hope, Lima, Peru; d Instituto Nacional de Ciencias Neurologicas, Lima, Peru; e Department of Neurology and Neurological Sciences, Stanford University, Palo Alto, CA, United States

**Keywords:** Hashimoto’sencephalopathy, Superrefractorystatusepilepticus, Autoimmunethyroiditis, Responsiveencephalopathy, Immunotherapy

## Abstract

Hashimoto’s encephalopathy (HE), also known as steroid responsive encephalopathy associated with autoimmune thyroiditis (SREAT), is a rare autoimmune disorder characterized by neuropsychiatric manifestations and a favorable response to corticosteroids. Seizures occur in nearly half of patients; however, super refractory status epilepticus (SRSE) is an uncommon initial presentation. We describe a young adult woman with Hashimoto’s thyroiditis who developed SRSE after a brief prodrome of headache, confusion, and paranoia. Extensive infectious, metabolic, and autoimmune workup was negative, but anti-thyroid peroxidase and anti-thyroglobulin antibodies were markedly elevated. Brain MRI showed nonspecific subcortical T2/FLAIR hyperintensities. The patient was diagnosed with HE and treated with high dose intravenous methylprednisolone followed by intravenous immunoglobulin, resulting in full neurological recovery and sustained seizure freedom at three-month follow up. This case illustrates the importance of considering HE in patients with unexplained SRSE, as prompt recognition and immunotherapy can be lifesaving.

## Highlights

• Super refractory status epilepticus may be an initial presentation of SREAT.
• Elevated antithyroid antibodies support the diagnosis of Hashimoto’s encephalopathy.
• Seizures in SREAT may be resistant to antiepileptic drugs and steroids alone.
• Combined corticosteroids and IVIG can lead to full neurological recovery.

## 1 Introduction

Hashimoto’s encephalopathy (HE), also known as Steroid Responsive Encephalopathy Associated with Autoimmune thyroiditis (SREAT), is a rare autoimmune neurologic disorder with an estimated prevalence of 2 per 100,000 individuals. The average age of onset is between 45 and 55 years, and the female-to-male ratio of approximately 5:1 [1]. Seizures are reported in almost half of patients with SREAT. However, initial presentation as super refractory status epilepticus (SRSE) is exceptionally uncommon, with only isolated cases reported in the literature. We present this case to illustrates the importance of considering SREAT in patient with unexplained SRSE.

## 2 Case history

A 27 year old woman with a history of Hashimoto’s thyroiditis (on levothyroxine 100  µg daily) and major depressive disorder (on sertraline 100  mg daily) presented with a five-day history of left-sided headache, followed by intermittent episodes of confusion and paranoia. Shortly before admission, she experienced a generalized tonic-clonic seizure and lost consciousness for approximately one hour. During transport to the hospital, was treated with intravenous diazepam. Upon arrival, she was alert, oriented, and exhibited no focal neurological deficits. Initial laboratory tests, including complete blood count, metabolic panel, and inflammatory markers, were within normal limits.

**Fig. 1:**
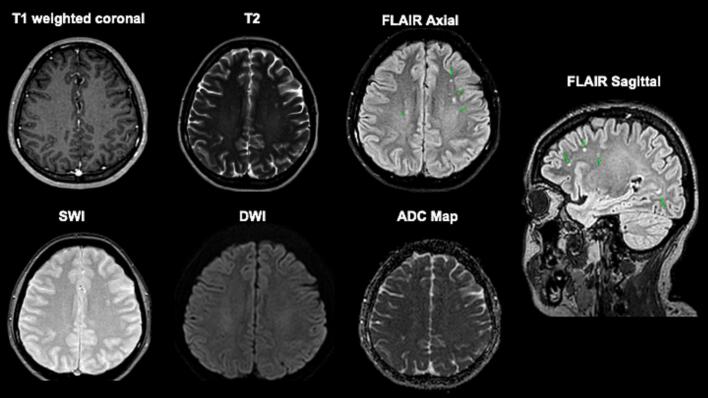
Electroencephalogram during ICU stay showing generalized slowing, consistent with diffuse encephalopathy, and rare sharp waves in the left frontal region (highlighted by red circle), in keeping with scarce epileptiform activity. (For interpretation of the references to colour in this figure legend, the reader is referred to the web version of this article.)

During hospitalization, she developed recurrent seizures progressing to refractory status epilepticus, requiring transfer to the intensive care unit, endotracheal intubation, and intravenous phenytoin administration. EEG demonstrated generalized slowing pattern suggestive of encephalopathy, along with sparse epileptiform discharges (Fig. 1).

**Fig. 2:**
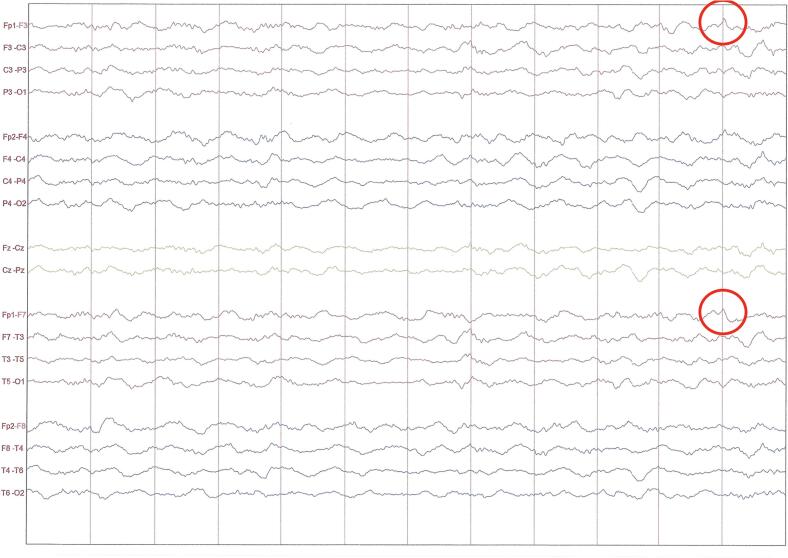
Axial T1-weighted coronal image (top left), T2-weighted (top middle), FLAIR (top right, green arrows), susceptibility-weighted imaging (SWI; bottom left), diffusion-weighted imaging (DWI; bottom middle), and apparent diffusion coefficient (ADC) map (bottom right) sequences. Images demonstrate adequate differentiation between gray and white matter, with no evidence of acute infarction, hemorrhage, or mass effect. Several ovoid hyperintensities are observed on T2/FLAIR sequences, located in the subcortical white matter of the centrum semiovale and corona radiata in both frontoparietal lobes, sparing juxtacortical fibers. No diffusion restriction is noted on DWI, and no abnormal contrast enhancement is seen following gadolinium administration. These hyperintense lesions are likely related to cerebral microvasculature involvement or small vessel disease (microangiopathy). (For interpretation of the references to colour in this figure legend, the reader is referred to the web version of this article.)

Brain MRI revealed no acute pathology, supporting the absence of structural lesion (Fig. 2). Empirical antibiotics and antivirals were initiated but later discontinued following negative cerebrospinal fluid cultures, a multiplex PCR meningitis/encephalitis panel (FilmArray®, BioFire Diagnostics, USA) that ruled out bacterial, viral, and fungal pathogens, an autoimmune encephalitis antibody panel (Euroimmun®, Germany) that was negative for neuronal surface antibodies including NMDA, LGI1, CASPR2, AMPA, and GABA, and serological testing for syphilis and HIV were negative. Thyroid antibody testing revealed elevated anti-thyroglobulin (466.7  IU/mL) and anti-TPO (344.1  IU/mL) antibodies, with normal TSH and free T4 levels. ANA was positive (1:100, speckled pattern), while ENA panel and complement levels were normal. Based on the clinical and laboratory findings, we considered the diagnosis of Hashimoto’s encephalopathy.

High-dose IV methylprednisolone (1  g/day for 5 days) was administered, followed by IV immunoglobulin due to persistent seizures after the first extubation attempt. Gradual clinical improvement was observed, and the patient was successfully extubated. Maintenance therapy with oral corticosteroids and levetiracetam was initiated. Repeat EEG demonstrated resolution of epileptiform activity. She was discharged in stable condition and remained seizure free with preserved cognition at 3 months.

## 3 Discussion

SREAT, first described in 1996, is a rare syndrome of neuropsychiatric symptoms diagnosed mainly by excluding other causes and observing significant symptom improvement with corticosteroids [1]. The four main criteria for diagnosing are: 1) altered consciousness with cognitive changes; 2) new or worsening psychiatric symptoms; 3) elevated serum TPO antibodies (≥0.5 U/mL) or other antithyroid antibodies; and 4) exclusion of infectious, toxic, metabolic, or neoplastic causes [2]. Our patient met these criteria after exclusion of all alternative etiologies.

SREAT can present acute or subacute with seizures (47%), confusion (46%), speech disorder (37%), gait disturbance (27%) and psychosis or paranoia (25%). Status epilepticus (SE) occurs in 12% of patients with SREAT [3]. Our patient presented with super refractory status epilepticus, an extremely rare manifestation of SREAT. Only one similar case has been reported in the literature, in which the patient deteriorated despite treatment, ultimately resulting in death on day 18 of hospitalization [4]. Approximately 70% of patients with SREAT exhibit elevated titers of antithyroid antibodies, most commonly both TPO and anti-TG antibodies [5]. A reported case of complete symptom resolution after total thyroidectomy suggests a potential role of these antibodies in the pathogenesis and severity of the disease, though further research is needed [2]. EEG findings in SREAT typically demonstrate generalized slowing with focal temporal or frontal involvement, while MRI often reveals cerebral atrophy and increased T2/FLAIR white matter signals [1], [2], [3]. Given the lack of specific clinical, EEG, or MRI features for SREAT, testing for antithyroid antibodies is essential in patients with unexplained SRSE, as they remain a key component in reaching the diagnosis.

First-line therapy typically involves high-dose corticosteroids, achieving response rates of approximately 60% [3]. A commonly employed regimen consists of intravenous methylprednisolone (500–1000 mg daily for one week), followed by oral prednisone (1–2 mg/kg/day for six to eight weeks) with gradual tapering after clinical recovery [2], [6]. However, status epilepticus in the context of SREAT presents a therapeutic challenge due to its frequent resistance to both antiepileptic drugs and steroids [7]. In such cases, more intensive and combined immunomodulatory therapies such as intravenous immunoglobulin (IVIG), plasma exchange, or other immunosuppressive agents may be required to achieve clinical improvement [3], [8]. In our case, treatment with high-dose corticosteroids followed by IVIG led to complete recovery without relapse.

Current evidence indicates that while antibody levels and MRI findings are useful for initial diagnosis, they do not reliably correlate with disease severity. Consequently, in clinically stable patients, routine serological or radiological follow-up is not generally required, and close clinical monitoring remains the recommended approach [1], [2]. This is supported by data from larger series, which demonstrated that at a median follow-up of 12 months, 90% of patients achieved complete or partial neurological improvement, although approximately 15% experienced at least one relapse and 5% died due to complications such as infection, myocardial infarction, or myocarditis [3]. In our case, at three months, the patient remained clinically stable with no seizure recurrence and preserved cognitive function.

## 4 Ethics

I Testify on behalf of all co-authors that our article submitted followed ethical principles in publishing

## CRediT authorship contribution statement

**Andres Chaponan-Lavalle:** Writing – review & editing, Writing – original draft, Investigation, Formal analysis, Conceptualization. **Jorge Alave:** Writing – review & editing, Supervision, Formal analysis. **Fred Fernandez:** Writing – original draft, Investigation. **Luciana Chacon Hermoza:** Writing – original draft, Investigation. **Danny M. Barrientos-Iman:** Writing – original draft, Supervision, Investigation. **Roberto Chulluncuy-Rivas:** Writing – review & editing, Writing – original draft, Supervision, Investigation. **Nelson Diaz:** Writing – review & editing, Writing – original draft, Supervision, Investigation.

## Funding

No funding was received from this work.

## Declaration of competing interest

The authors declare that they have no known competing financial interests or personal relationships that could have appeared to influence the work reported in this paper.

## References

[1] Ercoli T, Defazio G, Muroni A. Status epilepticus in Hashimoto’s encephalopathy [Internet]. Seizure 2019;70:1–5.[cited 2025 Jun 1 ] Available from: https://linkinghub.elsevier.com/retrieve/pii/S1059131119302894. 10.1016/j.seizure.2019.06.020 31228700

[2] Adithya-Sateesh B., Gousy N., Gogna G. (2023). Encephalopathy of autoimmune origin: steroid-responsive encephalopathy with associated thyroiditis. AACE Clin Case Rep.

[3] Laurent C, Capron J, Quillerou B, et al. Steroid-responsive encephalopathy associated with autoimmune thyroiditis (SREAT): Characteristics, treatment and outcome in 251 cases from the literature [Internet]. Autoimmunity Reviews 2016;15(12):1129–1133.[cited 2025 Jun 1 ] Available from: https://linkinghub.elsevier.com/retrieve/pii/S1568997216302038. 10.1016/j.autrev.2016.09.008 27639840

[4] Al-Busaidi M, Burad J, Al-Belushi A, Gujjar A. Super Refractory Status Epilepticus in Hashimoto’s Encephalopathy [Internet]. Oman Med J 2017;32(3):247–250.[cited 2025 Aug 31 ] Available from: http://omjournal.org/articleDetails.aspx?coType=1&aId=1960. 10.5001/omj.2017.46 PMC5447801 28584608

[5] Croce L., Dal Molin M., Teliti M., Rotondi M. (2024). Hashimoto’s encephalopathy: an endocrinological point of view [internet]. Front Endocrinol.

[6] Chaudhuri J, Mukherjee A, Chakravarty A. Hashimoto’s Encephalopathy: Case Series and Literature Review [Internet]. Curr Neurol Neurosci Rep 2023;23(4):167–175.[cited 2025 Jun 1] Available from: https://link.springer.com/10.1007/s11910-023-01255-5. 10.1007/s11910-023-01255-5 PMC9972331 36853554

[7] Khatib S., Jaber F., Elsayed M. (2022). Hashimoto’s thyroiditis presents as an acute encephalopathy: a case report of unusual presentation [internet]. Cureus.

[8] Şorodoc V., Constantin M., Asaftei A. (2023). The use of intravenous immunoglobulin in the treatment of Hashimoto’s encephalopathy: case based review [internet]. Front Neurol.

